# Supplementary material for: Preventive and treatment efficiency of dendrosomal nano-curcumin against ISO-induced cardiac fibrosis in mouse model
Source: PLoS One. 2024 Oct 10;19(10):e0311817. doi: 10.1371/journal.pone.0311817 (PMC11469592; doi:10.1371/journal.pone.0311817)
Supplement: S1 Table — (DOCX) [file pone.0311817.s001.docx]

**Table 1** Primer sequences used for RT-qPCR analysis.

| **Gene** | **Forward primers (5'-3')** | **Reverse primers (5'-3')** |
| --- | --- | --- |
| B2M | TGGTCTTTCTGGTGCTTGTC | TATGTTCGGCTTCCCATT CTC |
| COL1A1 | ATGGATTCCCGTTCGAGTACG | TCAGCTGGATAGCGACATCG |
| COL3A1 | GACCAAAAGGTGATGCTGGACAG | CAAGACCTCGTGCTCCAGTTAG |
| TGF-β1 | ATCCTGTCCAAACTAAGGCTCG | ACCTCTTTAGCATAGTAGTCCGC |
| SMAD3 | AGTGCATTACCATCCCCAGG | AGGAGGTGGGGTTTCTGGAA |
| SMAD2 | TGCTCTTCTGGCTCAGTCTG | CTGCCTCCGATATTCTGCTCC |
| MMP-9 | GCGTGTCTGGAGATTCGACT | CTTGGTACTGGAAGATGTCGT |
| FURIN | GCTGACCAAGTTCACCCTCG | AAGCCTTCCTCGCACACCAC |
| a-SMA | GAGGCACCACTGAACCCTAA | CATCTCCAGAGTCCAGCACA |
| COMP | GGAGTCCCTAACGAGCAAGAC | CCATCTATGTCGTCGTCGCAG |
| MYH11 | GCAACTACAGGCTGAGAGGAAG | TCAGCCGTGACCTTCTCTAGCT |
