# Supplementary figures and images for: Preventive and treatment efficiency of dendrosomal nano-curcumin against ISO-induced cardiac fibrosis in mouse model
Source: PLoS One. 2024 Oct 10;19(10):e0311817. doi: 10.1371/journal.pone.0311817 (PMC11469592; doi:10.1371/journal.pone.0311817)

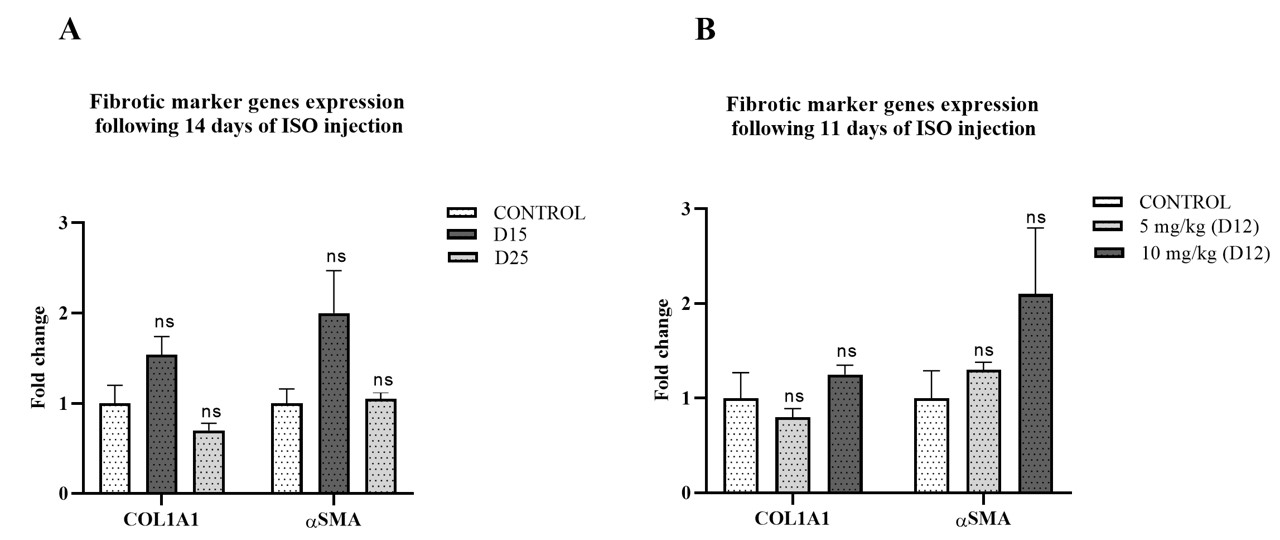

Supplement: S1 Fig — (A) The expression of COL1A1 and α-SMA genes was evaluated through RT-qPCR after ISO injection (10 mg/kg for 3 days and 5 mg/kg for 11 days) with mice being harvested on day 15 and day 25 compared to the control group. (B) The mRNA level of COL1A1 and α-SMA genes in ISO (5 mg/kg and 10 mg/kg for 11 days) treated mice was determined by RT-qPCR compared to the control group. Data are presented as mean ± SEM vs control (n = 3). The mean expression was shown as a fold change. T Student’s t-test: non-significance. (TIF) [file pone.0311817.s002.tif]

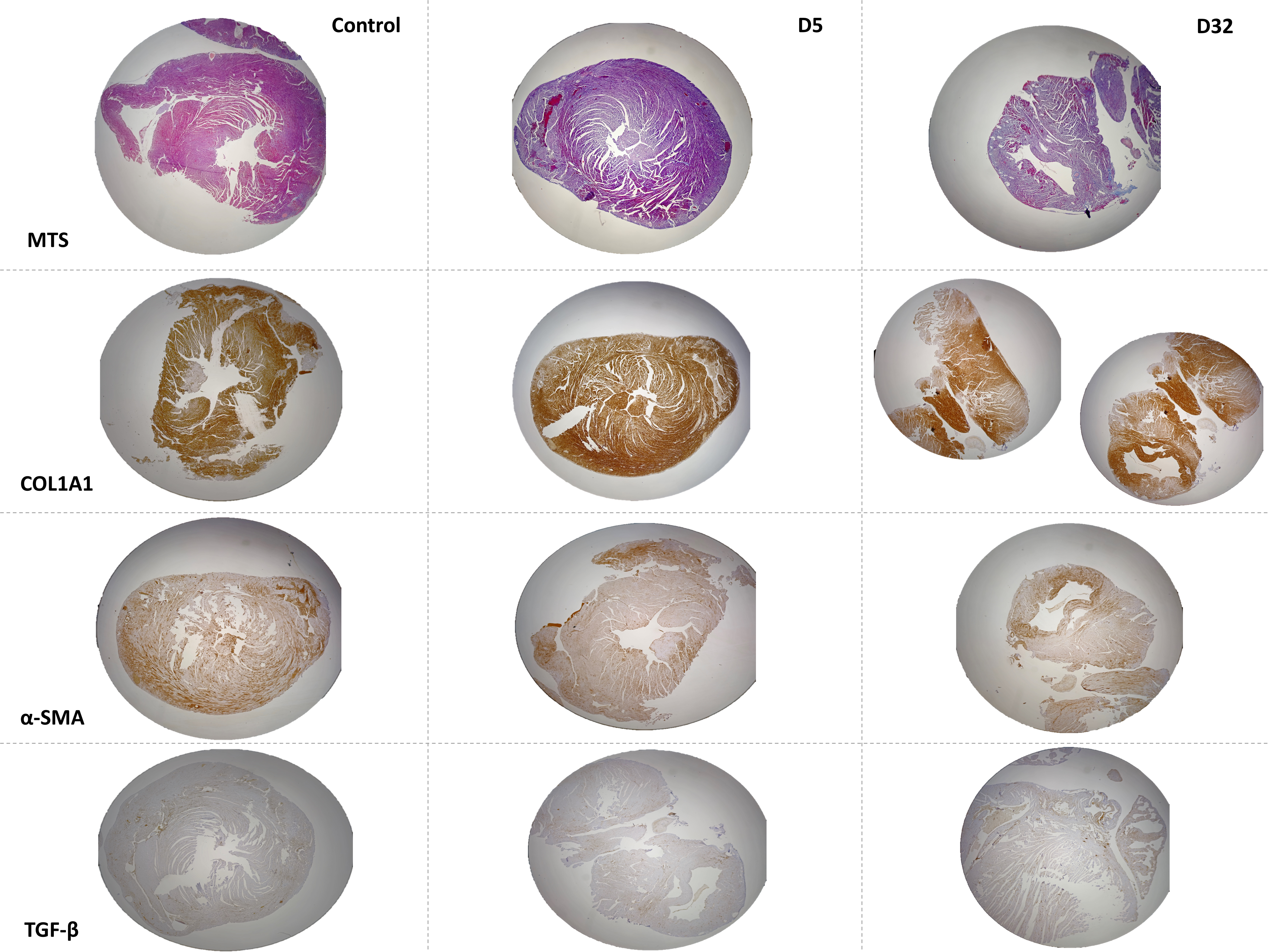

Supplement: S2 Fig — Complete images of heart tissue for MTS and IHC for markers COL1A1, α-SMA, and TGF-β were taken using a 4x microscope lens. (TIF) [file pone.0311817.s003.tif]
