# Supplementary material for: Preventive and treatment efficiency of dendrosomal nano-curcumin against ISO-induced cardiac fibrosis in mouse model
Source: PLoS One. 2024 Oct 10;19(10):e0311817. doi: 10.1371/journal.pone.0311817 (PMC11469592; doi:10.1371/journal.pone.0311817)
Supplement: S1 File — (DOCX) [file pone.0311817.s004.docx]

**Choosing the injection type to induce pulmonary fibrosis**

After doing protocol #1 (14 days injection of 10 & 5 mg/kg), RT-qPCR was used to evaluate the mRNA level of COL1A1 and αSMA genes as the well-known biomarkers of cardiac fibrosis induction (Fig 1. A and B) in both groups of mice injected subcutaneously and intraperitoneally. Upregulation of these genes was observed in intraperitoneal injection, although they were not significant compared to the control group. The intraperitoneal injection method was chosen over the subcutaneous injection method as it was more effective in inducing pulmonary fibrosis.

**A**

**B**


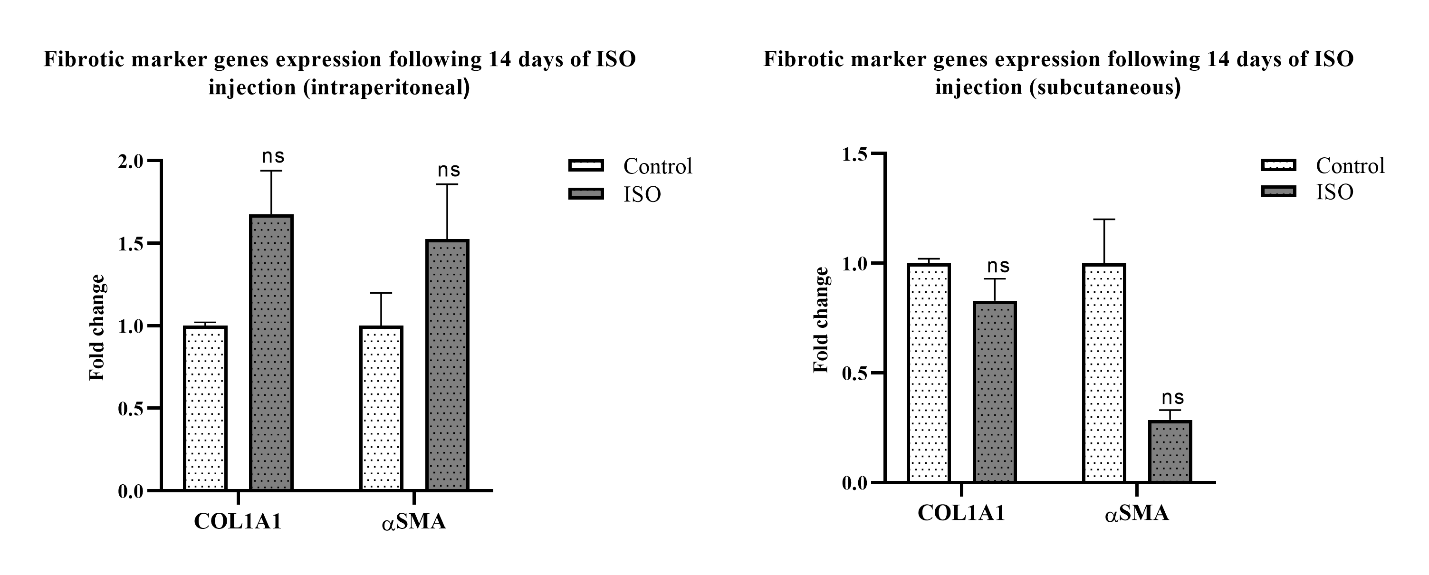


**Fig 1. Intraperitoneal injection is an effective method for inducing pulmonary fibrosis with ISO.** The expression of COL1A1 and α-SMA genes was evaluated through RT-qPCR after intraperitoneally (A) and subcutaneously (B) ISO injection (10 mg/kg for 3 days and 5 mg/kg for 11 days) in comparison to the control group. Data are presented as mean ± SEM vs. control (n = 3). The mean expression was shown as a fold change. T Student's t-test: non-significance.
