# Supplementary material for: Preventive and treatment efficiency of dendrosomal nano-curcumin against ISO-induced cardiac fibrosis in mouse model
Source: PLoS One. 2024 Oct 10;19(10):e0311817. doi: 10.1371/journal.pone.0311817 (PMC11469592; doi:10.1371/journal.pone.0311817)
Supplement: Attachment — Submitted filename: Response to Reviewers.docx [file pone.0311817.s005.docx]

**Response to Reviewers**

**Submission ID:** PONE-D-24-22014

**Manuscript Title:** Preventive and treatment efficiency of Dendrosomal Nano-Curcumin against ISO-induced cardiac fibrosis in mouse model

Dear Editor;

I would like to thank the reviewers for their careful reading of this manuscript and their suggestions and thoughtful comments which helped to improve the quality of this manuscript.

Our response follows:

**Reviewers' comment:** Authors must provide data validating that they have fibroblasts and not VSMCs or other cell types.

**Response:** We have used a well-established protocol reported in the manuscript (reference # [30]), to isolate fibroblast from the mouse cardiac mixture of cells. Accordingly, α-SMA and also other marker genes (vimentin and fibronectin) expression are used to differentiate fibroblasts from the other cells in the preparation. Along with molecular markers, morphology of the cells were used to support the identity of the fibroblasts (1). Similarly, references to methods from other studies have also been included. As stated in the manuscript, α-SMA expression was measured and reported because a key distinctive feature differentiating myofibroblasts from fibroblasts and other cardiac cells is the presence of α-SMA. The expression of α-SMA is the gold standard for identifying activated fibroblasts (2).

In details, cardiac fibroblast cell culture has been performed globally for many years, with established protocols. In the final stage, after 3 hours, fibroblasts adhere to the plate while other cells are removed. Numerous articles on cardiac fibrosis have been published using primary cardiac fibroblast cell cultures without any tests to confirm their fibroblast nature (3-5). **However, despite repeating this procedure, we used molecular testing for confirmation. This content is placed in the section Primary Cardiac Fibroblast Culture (Page 4)**.

**Reviewers' comment:** Regarding histology: Authors should show the entire heart on the slide with XY stitching to confirm that the representative image reflects a similar degree of fibrosis between groups. Authors should also take the images at the same time with the same color contrast and white background adjustments to ensure that the color is the same between images so that they can be adequately assessed.

**Response:** Since the project has been accomplished, providing such figures means repeating all of the experiments. In order to address this issue, we supplemented our manuscript with images of some complete cardiac sections in the supplementary file (S2_Fig).

For COL1A1 on day 32, where two images are placed, they complement each other. Due to the lack of a Microscope Scanner, two images were taken side by side to show the complete image of the tissue section with a 4x lens.

In Figure 4, where images with 400x zoom were placed, these images were taken by a pathologist from cardiomyocytes and areas in the left ventricle of the heart that are involved in fibrosis. For example, we naturally have collagen expression in parts of the heart such as vascular walls, or α-SMA and TGF-β show normal expression in vascular walls in the control group and on day 5. Therefore, the images in Figure 4 were taken from abnormal areas such as cardiomyocytes and evaluated using ImageJ. The interpretation and explanation of this section are provided in the discussion part.

**Reviewers' comment:** What was the vehicle that DNC was in for cell treatment?

**Response:** Dendrosome was used as a nanocarrier for curcumin.

Nano-curcumin, a polymer synthesized using dendrosomes, not only increases biostability but also enhances its solubility and intracellular absorption. DNC, a curcumin-loaded dendrosome nanocarrier, has demonstrated high efficacy in drug delivery across various studies. It was developed at Tarbiat Modares University **(Discussion, page 14).** In the Materials and Methods section, under the MTT subsection, the purchase of this substance and its protocol are described, along with the provided reference.

**Reviewers' comment:** For cells, how was the ISO dose determined? Is there MTT data available for this compound?

**Response:** This dose ISO (10µm) has been widely used in various in vitro studies. Two references supporting the use of this dose have been added to the manuscript text **(Material and methods - ISO and DNC treatment, page 5, line 146).**

**Reviewers' comment:** The data presented clearly demonstrate that the fibrosis model (protocol #4; 140 mg/kg ISO) is unreliable, as the levels of fibrotic markers are inconsistently expressed when in fact they should be up-regulated if cardiac fibrosis is evident. Please explain the differential expression of these markers.

**Response:** If we just focus on the RT-qPCR results, there seems unexpected results of gene expression during the days 7th to 25th. However, looking at Fig.3 RT-qPCR analysis shows that TGFB, SMAD3, Furin, Col1A1, aSMA, MMP9 all have been upregulated in day 5th of the process, consistent to the MTS (Fig. 4). Also, TGFB, aSMA, and MMP9 expression are again upregulated in day 32 as the second wave of gene expression upregulation, consistent to the IHC (Fig. 4). As explained in the article, day 5 showed the beginning of collagen formation histologically, while on day 32, collagen fibers were visible in the left ventricle, and SMA changes were well-demonstrated through IHC. This indicates the persistence of fibrotic conditions even 28 days after the initiation of this process. We expected that on day 32, these changes would be more evident at the tissue level rather than at the RNA level, which was indeed the case.

Please take into the consideration that fibrosis is an extremely complex process and isoproterenol induces multiple types of cardiac fibrosis (replacement, interstitial, and perivascular)(6-8). Therefore, specific signaling pathways regulate each of these types, potentially creating a complex network of interactions and gene expression alterations(9). Consequently, during the intermediate days (D7, 11, 18, 25) when some genes show no change, they may be influenced by these diverse pathways. It's possible that during these days, genes outside the TGF-β pathway might exhibit significant expression changes. For instance, on day 32, TGF-β might have been activated through an alternative signaling pathway, which subsequently led to the observed increase in SMA expression. The crucial point is that we observed clear changes in these genes on day 5, indicating the induction of fibrosis. This observation is significant in demonstrating the onset of the fibrotic process. It is not clear why the upregulation of marker genes follows the double wave model of gene expression upregulation. Double wave of stress response is not unusual in different organisms including plant and animals.

There is no standardized dose for inducing fibrosis with ISO, and each study has used a different dose. However, a common feature among many studies is that they have used **the day immediately following isoproterenol injection** as the day to examine cardiac fibrosis. In our study, day 5 (**one day** after consecutive ISO injections) showed extensive expression changes in cardiac fibrosis markers. This indicates that day 5 is crucial for initiating the fibrosis process.

**(Discussion, Page 16, line 564-569)**

**Reviewers’ comment:** Can the authors explain/discuss the significance of their novel protocol in light of the different phases of fibrosis (inflammation, proliferation, and maturation phases)?

**Response:** According to the time/tissue serials of RT-qPCR investigation and also intensity of the fibrotic-markers staining, we came to the conclusion that regardless of fibrosis type, there could be two phases of cardiac fibrosis induction (day 5) and stabilization (day 32). Nevertheless, since isoproterenol is a compound capable of inducing all three models of cardiac fibrosis, distinguishing them is hard and it is feasible when we specifically induce and examine a single model of it.

In the discussion section of the article, based on the results obtained from this model on the two main days (5 and 32), we have discussed the early and late expression of these markers. Some markers showed a significant increase in expression only in the early days as an induction phase, while others showed increases in both early and late (day 32) stages (induction and stabilization phases). This pattern has been reported for both cardiac and pulmonary fibrosis.

**Reviewers’ comment:** Data in Fig 4d,g contradicts the data in Fig 3a and shows more Col1a1 is present on D32 vs D5.

**Response:** The initial upregulation of gene expression often rapidly responds to injury or stress, while the accumulation of protein and corresponding histological changes take longer to manifest. Proteins such as collagen have a longer half-life compared to RNA, thus even after the gene expression levels decline, the proteins can continue to accumulate and persist in the tissue (9-12). Therefore, in our study, the expression level of collagen had returned to its normal state by day 32, while the collagen fibers had matured histologically. Generally speaking, proteins can have much longer half-lives than mRNA, allowing them to accumulate even when transcription and translation have slowed (13).

**(Discussion, Page 16, line 582-588)**

An example from the study on Cx43 and NaV1.5 expression:

The observed reduction in Cx43 protein level correlated with downregulation of Gja1 RNA expression at postnatal weeks 1 and 2 in MHC-CnA. However, at weeks 3 and 4 the RNA level of Gja1 normalized to WT values, whereas the protein level remained reduced. This suggests that the initial Cx43 reduction is caused at the transcriptional level, whereas at weeks 3 and 4 another mechanism may be responsible (14).

**Reviewers’ comment:** Data in Fig 5 are very confusing and lack clear rationale and interpretation. The apparent upregulation of TGFb, MMP9 with ISO-DNC is problematic and indicates non-specific effect of DNC.

**Response:** For DNC injection in mice, we had four groups, which are schematically represented in this figure for better understanding.

The selection of DNC injections as a treatment modality was made for clinical purposes. Pre-treatment and co-treatment with DNC are employed to assess their effectiveness in preventing cardiac fibrosis. Furthermore, post-induction DNC treatment aims to explore its potential inhibitory effects on fibrosis progression and its therapeutic utility. Actually, we intended to mimic the heart attack (fibrosis induction) and DNC-treatment of it before and after the attack. Suppose that we have a drug called DNC and want to pre-treat or post- attack treating the patient with this drug.

Part A, mimics someone who is prescribed with DNC for 7 days before the induction and during the fibrosis induction. Results showed that pretreatment with DNC for 7 days, followed by co-treatment of ISO and DNC for 4 days (DNC/ISO+DNC) drastically reduces fibrosis marker genes expression immediately after the fibrosis induction (day 5^th^).

Part B, also mimics someone who is prescribed with DNC for 7 days before the induction and during the fibrosis induction. However, it measures the effect of DNC pretreatment and cotreatment on fibrosis progression in long term (day 32). This type of DNC-pretreatment also attenuated the fibrotic marker genes expression in long term (shown by column DNC/DNC+ISO sign).

If it is immediately applied after the heart attack (fibrosis induction), post-treatment of DNC is also effective for attenuating the fibrotic genes expression (shown by ISO-DNC (D5-D11) in the graphs).

However, if DNC is applied with delay (day 25^th^) after the heart attack (fibrosis induction by ISO), its protective effect won’t be reliable.

In another word, in the prevention group, notable reductions in the expression levels of COL1A1, α-SMA, and MMP9 were observed in mice sacrificed on Day 5, compared to those in the cardiac fibrosis group. Additionally, in mice exhibiting fibrosis on Day 32, wherein three markers (α-SMA, TGFβ, MMP9) were elevated, administration of DNC led to significant decreases in their expression levels.

In the second group, with the aim of inhibition and therapy, a decrease in the expression of the three mentioned markers was observed on D32. **However, when DNC was administered from D25 to D31, this reduction in expression was observed only in α-SMA. This indicates that** **post-treatment with DNC in the final days, specifically from day 25 to 31, cannot be very effective in reducing the expression of genes involved in fibrosis. The two markers, TGF-β and MMP9, did not show significant differences compared to the fibrosis group at day 32.** **DNC post-treatment should be administered immediately following isoproterenol use or, in medical terms, immediately after a heart attack to be effective.**

**(Discussion, Page 17, lines 612-614)**

**Reviewers’ comment:** Was there any evidence ISO triggers pulmonary fibrosis?

**Response:** Isoproterenol has not been used in any previous study to induce pulmonary fibrosis. As explained in the **discussion section**, bleomycin is typically used through an invasive method to induce this condition. We used isoproterenol for the first time to investigate the induction of pulmonary fibrosis. Important genes such as COL3, SMA, COL1, TGF-β, and MMP9 showed increased expression compared to the control group. However, unlike cardiac fibrosis where gene expression changes were observed on days 5 and 32, in pulmonary fibrosis, these genes showed a scattered expression pattern across different days.

This isoproterenol-induced pulmonary fibrosis model might be used in studies of cardiopulmonary fibrosis, if more decisive method of pulmonary fibrosis induction is not available.

**Reviewers’ comment:** The comparison of the RNAseq data should be interpreted with caution as the analyses were performed on completely different samples, human fibroblasts stimulated with TGFb in vitro and mice hearts subject to LAD ligation. It would have been conceivable had the authors have used RNAseq generated from human ischemic hearts (which are widely available) vs mice LAD. Also problematic is the stimulation times, 24hs in human vs days/weeks in mice. In addition, the RNAseq analysis is poorly explained and presented.

**Response:** In the bioinformatics section, our goal was to identify a gene that plays a role in every cardiac fibrosis induction model, including cell culture conditions. As mentioned, we have various methods for inducing cardiac fibrosis and can use different compounds. For this reason, we included different induction methods in this study. We also separately analyzed both human and mouse samples to find a common gene, so that after confirming its changes in mice, we could use it for investigation in humans as well. In fact, our objective was not to find a gene specific to one particular cardiac fibrosis induction model. Instead, we aimed to identify a gene that is universally involved across all conditions. This approach ensures that researchers can observe changes in this gene's expression regardless of the method used to induce fibrosis.

Given that we utilized samples from various studies that shared only cardiac fibrosis as a common factor, with different induction methods and species under investigation, all samples were not analyzed together or pooled. Initially, we analyzed the samples from each project separately and obtained the differentially expressed genes (DEGs) related to those samples.

However, to achieve our stated goal of finding a gene common to all these methods, we ultimately employed Venn analysis. By creating a Venn diagram, we were able to identify genes shared across all these studies. We visualized these identified common genes through a heat map. Additionally, we reported their molecular, biological, and cellular roles using functional enrichment analysis.

The interpretation of the enrichment results was also added to the manuscript. In addition to these aspects, details about the other tools used, such as STRING and Cytoscape, were added in the Methods and Results sections.

**(Materials and methods (Eligibility criteria and dataset selection), Page 7, line 245-250)**

**(Materials and methods (Finding hub genes), Page 8, lines 275-282)**

**(Results (Identification of DEGs), Page 12, lines 422-424)**

**(Results (Shared DEGs and enrichment analysis), Page 13, lines 435-451)**

**(Results (Protein-protein interaction network and hub genes analysis), Page 13, lines 458-464)**

**Reviewers’ comment:** How do the authors explain the fluctuation of COM expression in Fig8F?

**Response:** As explained earlier regarding other genes, COMP also showed a significant increase in expression on day 5, which is attributed to the rapid and initial response to isoproterenol and the onset of fibrosis induction. Moreover, as mentioned, isoproterenol can induce various types of fibrosis, each involving different mechanisms.

COMP, in addition to the TGF-β pathway, can be influenced by the Wnt/β-catenin pathway and pathways related to oxidative stress. Therefore, its significant increase on days 18 and 25 could be due to these other pathways. The key point is that this gene has shown a significant increase compared to the control on three different days.

Comments to the Author

1. Is the manuscript technically sound, and do the data support the conclusions?

The entire text of the manuscript was edited and the content that was asked by the reviewer was added to the text of the manuscript and the explanations of the results were presented in a transparent manner.

2. Has the statistical analysis been performed appropriately and rigorously?

As described in the statistical analyzes section of the manuscript, an unpaired two-tailed Student’s t-test was employed to compare the two groups. The one-way ANOVA was used to compare the means of more than two groups. A p-value of less than 0.05 was considered to define a statistically significant difference.

**References**

1.	Yousefi F, Soltani BM, Rabbani S. MicroRNA‑331 inhibits isoproterenol‑induced expression of profibrotic genes in cardiac myofibroblasts via the TGFβ/smad3 signaling pathway. Scientific Reports. 2021;11(1):2548.

2.	Ivey MJ, Tallquist MD. Defining the cardiac fibroblast. Circulation Journal. 2016;80(11):2269-76.

3.	Liu F, Chen Y, Qin D, Qian C. Interleukin-22 inhibits cardiac fibrosis by regulating fibroblast metabolic reprogramming in myocardial infarction. Pathology-Research and Practice. 2024;256:155256.

4.	Tan Y-m, Cao L-y, Jiao Y-q, Han L, Tang M-x, Wang Z-h, et al. Inhibition of miR-543 alleviates cardiac fibroblast-to-myofibroblast transformation and collagen expression in insulin resistance via targeting PTEN. Molecular and cellular endocrinology. 2023;576:111996.

5.	Hall C, Law JP, Reyat JS, Cumberland MJ, Hang S, Vo NT, et al. Chronic activation of human cardiac fibroblasts in vitro attenuates the reversibility of the myofibroblast phenotype. Scientific Reports. 2023;13(1):12137.

6.	Benjamin IJ, Jalil JE, Tan L, Cho K, Weber KT, Clark WA. Isoproterenol-induced myocardial fibrosis in relation to myocyte necrosis. Circulation research. 1989;65(3):657-70.

7.	Flori L, Lazzarini G, Spezzini J, Pirone A, Calderone V, Testai L, et al. The isoproterenol-induced myocardial fibrosis: A biochemical and histological investigation. Biomedicine & Pharmacotherapy. 2024;174:116534.

8.	Li N, Zhou H, Ma Z-G, Zhu J-X, Liu C, Song P, et al. Geniposide alleviates isoproterenol-induced cardiac fibrosis partially via SIRT1 activation in vivo and in vitro. Frontiers in Pharmacology. 2018;9:854.

9.	Frangogiannis NG. Cardiac fibrosis: Cell biological mechanisms, molecular pathways and therapeutic opportunities. Molecular aspects of medicine. 2019;65:70-99.

10.	Travers JG, Kamal FA, Robbins J, Yutzey KE, Blaxall BC. Cardiac fibrosis: the fibroblast awakens. Circulation research. 2016;118(6):1021-40.

11.	Ma Z-G, Yuan Y-P, Wu H-M, Zhang X, Tang Q-Z. Cardiac fibrosis: new insights into the pathogenesis. International journal of biological sciences. 2018;14(12):1645.

12.	Zhang Y, Stefanovic B. LARP6 meets collagen mRNA: specific regulation of type I collagen expression. International journal of molecular sciences. 2016;17(3):419.

13.	Lodish HF. Molecular cell biology: Macmillan; 2008.

14.	Fontes MS, Raaijmakers AJ, van Doorn T, Kok B, Nieuwenhuis S, van der Nagel R, et al. Changes in Cx43 and NaV1. 5 expression precede the occurrence of substantial fibrosis in calcineurin-induced murine cardiac hypertrophy. PloS one. 2014;9(1):e87226.
