# Supplementary material for: Preventive and treatment efficiency of dendrosomal nano-curcumin against ISO-induced cardiac fibrosis in mouse model
Source: PLoS One. 2024 Oct 10;19(10):e0311817. doi: 10.1371/journal.pone.0311817 (PMC11469592; doi:10.1371/journal.pone.0311817)
Supplement: Attachment — Submitted filename: Response to Reviewers.docx [file pone.0311817.s006.docx]

**Response to Reviewers**

**Submission ID:** PONE-D-24-22014

**Manuscript Title:** Preventive and treatment efficiency of Dendrosomal Nano-Curcumin against ISO-induced cardiac fibrosis in mouse model

Dear Editor;

I want to thank the reviewers for their careful reading of this manuscript and their suggestions and thoughtful comments which helped to improve the quality of this manuscript.

Our response follows:

**Reviewers' comment:** Authors need to verify the absence of MYH11 and smoothelin, vsmc-specific markers, to assess the purity of the cells. While PCR would suffice, flow cytometry would be ideal.

**Response**: Unfortunately due to the financial limitations and sanctions against our country, providing specific antibodies against MYH11, and Smoothelin to do flow cytometry is challenging and too expensive. Due to these limitations, we were only able to acquire primers for MYH11 to conduct PCR testing.

For our cell culture experiments, we used the heart tissue without the aorta, which significantly reduced the presence of VSMCs chance. Consequently, this method helped us to minimize the VSMC population in the initial cell cultures. Additionally, as the cells were passaged, the purity of fibroblasts in the culture increased, since VSMCs tend to be outcompeted in such conditions.

We also conducted an RT-PCR test against MYH11 expression on our cultured cells. As is shown in the attached figure, no MYH11 gene-related band was observed, indicating the **absence or non-detectable number of VSMCs** in our cultured cell preparation. Noteworthy, we used mouse lung tissue as a positive control of the RT-PCR reaction, where MYH11 is consistently expressed in the smooth muscle cells of the pulmonary vasculature and airways.

**The MYH11 primer sequences have been added to the table of primer sequences for use in RT-PCR testing. The negative result of the MYH11 PCR test has been added to the Materials and Methods section of the manuscript.**
